# Access to Essential Cardiovascular Medicines in Pakistan: A National Survey on the Availability, Price, and Affordability, Using WHO/HAI Methodology

**DOI:** 10.3389/fphar.2020.595008

**Published:** 2021-01-25

**Authors:** Amna Saeed, Faria Saeed, Hamid Saeed, Zikria Saleem, Caijun Yang, Jie Chang, Minghuan Jiang, Mingyue Zhao, Muhammad Saqlain, Wenjing Ji, Muhammad Majid Aziz, Krizzia Lambojon, Ali Hassan Gillani, Khezar Hayat, Sabiha Gul, Yu Fang, Zaheer-Ud-Din Babar

**Affiliations:** ^1^ Department of Pharmacy Administration and Clinical Pharmacy, School of Pharmacy, Xi'an Jiaotong University, Shaanxi, China; ^2^ Center for Drug Safety and Policy Research, Xi'an Jiaotong University, Shaanxi, China; ^3^ Shaanxi Centre for Health Reform and Development Research, Shaanxi, China; ^4^ Faculty of Pharmacy, the University of Lahore, Lahore, Pakistan; ^5^ Allama Iqbal Medical College, Lahore, Pakistan; ^6^ Department of Pharmaceutics, University College of Pharmacy, University of the Punjab, Lahore, Pakistan; ^7^ Department of Pharmacy, Quaid e Azam University, Islamabad, Pakistan; ^8^ Institute of Pharmaceutical Sciences, University of Veterinary and Animal Sciences, Lahore, Pakistan; ^9^ Department of Pharmacology, Faculty of Pharmacy, Hamdard University, Karachi, Pakistan; ^10^ Department of Pharmacy, School of Applied Sciences, University of Huddersfield, Huddersfield, United Kingdom

**Keywords:** cardiovascular drugs, access to medicines, medicines policy, essential medicines, non-communicable diseases

## Abstract

**Objective:** This national survey was aimed at measuring the access to cardiovascular disease (CVD) medicines in terms of their availability, price, and affordability in Pakistan. This was done by using the standard WHO/Health Action International (HAI) methodology.

**Methods:** The price and availability data for 18 CVD medicines were collected from public sector hospitals (n = 40) and private sector retail pharmacies (n = 40) in eight cities of Pakistan. The outcome measures were availability (calculated as percentage of health facilities stocked with listed medicines), medicine price to the international reference price ratio (i.e., median price ratio (MPR)), and affordability (calculated as number of days’ wages (NDWs) of the lowest paid unskilled government worker required to afford one-month treatment of a chronic disease). The affordability of standard treatment in Pakistan with four CVD drugs was compared with data from six other low and middle income countries (LMICs) using HAI database.

**Findings:** The mean percent availability of CVD medicines was significantly low (*p* < 0.001) in the public sector as compared to the private sector, that is, 25.5% vs. 54.6% for originator brands (OBs) and 30.4% vs. 34.9% for lowest price generics (LPGs), respectively. For all OBs and LPGs, the inflation-adjusted mean MPR was 2.72 and 1, respectively. CVD medicines were found to be unaffordable with average NDWs of 6.4 and 2.2 for OBs and LPGs, respectively, that is, NDWs of more than 1. In international comparison with countries such as Sudan, Lebanon, Egypt, India, Afghanistan, and China, the affordability of standard treatment with selected CVD medicines (atenolol, amlodipine, captopril, and simvastatin) in Pakistan was found to be low. Overall, all four OBs and three out of four LPGs of selected CVD drugs were found unaffordable in Pakistan.

**Conclusion:** This data indicated that the availability of selected CVD medicines was low in both public and private sector medicine outlets. Both OBs and LPGs were found unaffordable in the private sector, necessitating the redressal of pricing policies, structuring, and their implementation.

## Introduction

Globally, non-communicable diseases (NCDs) have emerged as a leading cause of mortality. These account for approximately 41 million people annually, which is equivalent to 71% of all the deaths worldwide (WHO, 2011). More than 85% of these deaths occur in low and middle income countries (LMICs). With population of 197 million, Pakistan is the fifth most populous country in the world, and about half of its population is suffering from one or more chronic diseases (Jafar et al., 2013; Population, Pakistan Bureau of Statistics, 2020). In 2013, Jaffer et al. estimated that in Pakistan, from 2010 to 2025, almost 3.87 million people will die due to NCDs, such as cardiovascular diseases (CVDs), cancers, and chronic respiratory diseases. The authors also projected that the economic burden associated with NCD deaths will be between $152 million and $296 million between 2010 and 2025 (Jafar et al., 2013). In Pakistan, the total health expenditure, as a percentage of the GDP, was 1.1% in 2018–2019. The public sector bears 32% of the health expenditures, while 64% is borne by patient's out of pocket (OOP) payments that may lead to catastrophic consequences for the families (Quick et al., 2002; Hsu et al., 2018; Khan, 2019; Ministry of Finance, Government of Pakistan, 2020). In public sector hospitals, the medicines are provided free of charge, while in private sector hospitals and retail pharmacies the patients have to pay out of pocket to obtain medicines.

Among NCDs, cardiovascular diseases (CVDs) pose a major threat with annual casualties of around 17.9 million (Lozano et al., 2012). According to the World Health Organization (WHO), CVDs include hypertension (HTN), coronary heart disease, cerebrovascular disease (stroke), peripheral vascular disease, heart failure, rheumatic heart disease, congenital heart disease, and cardiomyopathies (WHO, 2020a). According to the global burden of diseases data, CVDs are among the top ten leading causes of deaths in Pakistan with ischemic heart disease (IHD) leading the numbers (causing 8% of all deaths) (WHO, 2011; Lozano et al., 2012; Murray and Lopez, 2017).

The WHO recommends multi-drug therapy for the treatment and prevention of CVDs in patients with ≥30% risk of developing stroke or heart attack within ten years. These therapies include blood pressure (BP) lowering medicines, anti-hyperlipidemic (HLD) medicines, blood glucose control for diabetic patients, and anti-platelet drugs for secondary prevention of myocardial infarction. Despite strong clinical evidence about the effectiveness of medicines in preventing and controlling CVDs, disparity exists between the patients requiring treatment and those having actual access to these medicines (Wirtz et al., 2016). Moreover, a long-term therapy is required for NCDs, which incurs significant health care cost. For example, in 2010, Bloom et al. estimated the direct medical costs of 5 groups of NCDs (CVDs, diabetes, cancer, chronic respiratory diseases, and neurological disorders) to be US $3,705 billion, globally (The Global Economic Burden of Non-Communicable Diseases, World Economic Forum). A substantial portion of such medical costs of NCDs comprises medicines, making it obligatory to make the medicines affordable and accessible for patients (Mendis et al., 2005). Therefore, a Global Action Plan has been developed by the WHO aiming for 80% availability of affordable essential medicines for NCDs in both public and private health facilities (Beran et al., 2014). Moreover, “access to essential medicines” is a fundamental human right, and it has officially been declared as a pledge under Millennium Development Goal (MDG) 8, suggesting that the provision of affordable, high quality, and appropriate essential medicines is one of the major components of functioning health systems, which however remains questionable in LMICs (Sachs and McArthur, 2005; Zaidi et al., 2013). Besides, National Health Vision 2016–2025 was developed and aligned with Sustainable Development Goals (SDGs) to attain resilient and responsive health system. The notable institutional arrangement for health-related SDGs entails several vertical programs running at federal and provincial levels, such as malaria control, TB control, maternal and child health program, and HIV/AIDS control programs, along with the provision of medical staff, equipment, and essential medicines in all hospitals. However, the availability, affordability, accessibility, acceptability, and quality of medicines are the various key requisites to recuperate the health system in line with SDGs (Penchansky and Thomas, 1981; Pakistan Country Report-Sustainable Development Goals, 2017).

WHO-PREMISE (Prevention of Recurrences of Myocardial Infarction and Stroke) study was conducted, predominantly in LMICs. The study assesses the use of medicines in the secondary prevention of CVDs. The study analyzed whether the patients received the prescribed therapy in 10 LMICs. The data revealed that only 48% of chronic heart disease (CHD) patients received beta blockers, 40% of CHD patients and 38% of stroke patients received ACE inhibitors, and 30% of CHD patients and 14% of stroke patients could receive statins (Mendis et al., 2005). Another study called WHO-PURE (Prospective Urban Rural Epidemiology) was conducted to estimate the availability and affordability of four therapeutic classes of CVD medicines in 18 countries. The study findings suggested that the availability of CVD medicines was only 62% in urban community (UC) and 37% in rural community (RC) in lower middle income countries, and it was 80% in UC and 73% in RC in upper middle income countries, and it decreased to only 25% in UC and 3% in RC in low income countries. The treatment with CVD medicines was unaffordable to 25%, 33%, and 60% of the households in the upper middle income, lower middle income, and low income countries, respectively (Khatib et al., 2016).

Studies, conducted in Ghana, Brazil, and Mexico, reported that high medicine prices are major contributors that may lead to reduced patient adherence to medication therapy and hence can compromise the access to CVD medicines (Calvo-Vargas et al., 1998; Buabeng et al., 2004; De Tole̊do Nóbrega et al., 2007). However, many of these studies did not use standardized methodologies. In this context, WHO and Health Action International (HAI) developed a standardized methodology, and more than 100 surveys have been conducted by using this methodology. This is a valid method to measure the access to medicines in terms of availability and affordability and can also be used for a reliable comparison of access to medicines among different countries (WHO, 2015). In 2010, a secondary data analysis of WHO/HAI methodology surveys assessed the access to CVD medicines in 36 countries, and the availability was found to be low. It was observed to be 26% in public sector and 57% in private sector medicine outlets for a number of medicines. It was also reported that the treatment for CVD was not affordable in many countries, especially LMICs (van Mourik et al., 2010). Moreover, WHOs’ Global Action Plan has set a major goal to achieve 50% use of recommended CVD medications worldwide by 2025. Thus, widespread and capacious availability and affordability of these medicines are necessary to attain this goal (WHO, 2013).

About fifteen years ago, the first WHO/HAI survey of Pakistan was conducted by Kiani et al. (2006) to evaluate the access of 29 essential medicines and reported poor availability and affordability of these medicines in the region. After this, the WHO/HAI methodology was improved and updated in 2008 (WHO, 2015). In 2016–2017, another WHO/HAI survey (following updated methodology) to evaluate the access to 50 essential medicines in general was conducted in Lahore division in Pakistan (Saeed et al., 2019). A subsequent survey was performed by Saeed et al. in 2019, using the same 50 drugs to evaluate the impact of new National Drug Pricing Policy (NDPP) 2018 on the access to medicines and compare their prices, availability, and affordability in both years, that is, 2017 and 2019. The authors reported a significant increase in drug prices with slight improvements in the availability of these drugs, yet considerably poor in both years, that is, far less than 80% (Saeed et al., 2019). However, these surveys were conducted in only one administrative unit of Pakistan, that is, Lahore division, and the results could not be generalized to the whole of Pakistan. Besides, from Pakistan, to our knowledge, not a single study has been conducted focusing on a specific class of drugs or diseases; that is, only a few drugs were included from each category.

So, this is the first national scale study in Pakistan to measure the access to CVD medicines in the country. Since this study covers the major regions of Pakistan, it would also give an insight into the regional disparities in access to CVD medicines. In this context, the objective of this study was to evaluate the availability, price, and affordability of CVD medicines in Pakistan and to compare the local situation with other LMICs using standard WHO/HAI methodology.

## Methodology

### Study Outline

A cross-sectional survey was conducted by using standard WHO/HAI methodology. The data on 18 selected CVD medicines’ availability, price, and affordability were collected from September to December, in 2019. A total of eight regions/cities were selected for the survey, that is, Islamabad (federal capital), Lahore (Punjab province), Bahawalpur (Punjab province), Peshawar (Khyber Pakhtun Khwa (KPK) province), Abbottabad (KPK province), Karachi (Sindh province), Quetta (Baluchistan province), and Muzaffarabad (Azad Jammu and Kashmir (AJK) autonomous region). Thus, all four provinces of Pakistan, one of the two autonomous regions (i.e., AKJ), and the federal capital were included in the survey (see Table 1). Trained data collectors gathered the data on availability and patient prices of all selected medicines from both public sector hospitals and private retail pharmacies.

**TABLE 1:** Number of outlets samples and geographical location of participating cities.

Province	Provincial/regional population	City surveyed	Number of facilities in each city			
			Tertiary care hospitals	Secondary care hospitals	Primary healthcare centers	Private retail pharmacy
Federal capital	1.015 million	Islamabad	2	2	1	5
Punjab	110 million	Lahore (provincial capital)	3	2	0	5
		Bahawalpur	1	3	2	5
Khyber Phakhtoon Khuwa (KPK)	35.53 million	Abbottabad	1	2	2	5
		Peshawar (provincial capital)	1	2	2	5
Sindh	47.9 million	Karachi (provincial capital)	2	3	0	5
Baluchistan	12.34 million	Quetta (provincial capital)	1	3	1	5
Azad Jammu and Kashmir (AJK) (special administrative region)	4.45 million	Muzaffarabad	1	2	2	5

### Sampling Plan

The WHO/HAI methodology (details explained in the WHO/HAI survey manual) recommends to survey a minimum of six survey areas in a survey region (WHO, 2015). In this study, we increased the number of survey areas to eight, as Pakistan is a large country in terms of population. With the population of 197 million, Pakistan is the fifth most populous country in the world and belongs to the group of lower middle income countries, according to the World Bank (World Bank Country and Lending Groups, World Bank Data Help Desk, 2020). It consists of four provinces (Punjab, KPK, Sindh, and Baluchistan) and two autonomous regions (Gilgit-Baltistan and AJK). All provincial capitals and capital of an autonomous region along with federal capital were included in the study to make a representative sample of the whole country (see Table 1). Other than the capital city in each province, one additional city was included from the Punjab and KPK provinces.

As required by the standard WHO/HAI methodology, one biggest public sector hospital was selected as a survey anchor in each survey area. Four public sector hospitals within 3 km range from the survey anchor were randomly selected in each survey area, making a total of five public sector hospitals in each city/survey area. One private retail pharmacy was selected, within 5 km range of each public sector facility, making a total of five retail pharmacies in a survey area (see Supplementary Figure S1). In total, 80 medicine outlets were surveyed including 40 public sector hospitals and 40 retail pharmacies.

### Selection of Medicines

In this survey, 18 medicines used to treat CVDs were selected that included medicines from both global core list (n = 3) and supplementary list (n = 15). The global core list of medicines is provided in the WHO/HAI manual. It contains the essential medicines which are preferentially used while conducting all such surveys, which helps in performing international comparisons. According to the standard WHO/HAI methodology, each country can select supplementary medicines for survey on the basis of their significance and relevance in treating national healthcare problems (WHO, 2015). In this study, the supplementary medicines were carefully selected following the recommendations of WHO/HAI advisor (author ZB) and a national advisory board that consisted of physicians, pharmacists, drug procurement officers, and academicians. The National Essential Medicines List 2018 (NEML) was also consulted while selecting the medicines (Drug Regulatory Autority of Pakistan, 2018b). In this respect, 14 out of 18 selected medicines were also part of NEML 2018 (see Table 2).

**TABLE 2:** List of medicines for survey.

Medicine with strength and dosage form	Part of NEML (yes/no)	Indication/s for use	Pharmacological class
Furosemide 40 mg cap/tab	Yes	Hypertension and fluid retention in heart failure	Loop diuretic
Atenolol 50 mg tab	Yes	Hypertension	Beta blocker
Simvastatin 20 mg cap/tab	Yes	Hypercholesterolemia/prevention of CVDs	HMG-CoA reductase inhibitors
Amlodipine 5 mg tab	Yes	Hypertension	Calcium channel blocker
Captopril 25 mg cap/tab	No	Hypertension and congestive heart failure	ACE inhibitor
Enalapril 5 mg tab	Yes	Hypertension and prevention of symptomatic heart failure	ACE inhibitor
Nifedipine Retard 20 mg tab	No	Hypertension and prophylaxis of chronic stable angina pectoris	Calcium channel blocker
Atorvastatin 20 mg cap/tab	No	Hypercholesterolemia/prevention of CVDs	Lipid modifying agent
Hydrochlorothiazide 25 mg cap/tab	Yes	Hypertension	Diuretic
Digoxin 0.25 mg tab	Yes	Congestive heart failure	Cardiac glycoside
Losartan 50 mg cap/tab	Yes	Hypertension and stroke prevention	Angiotensin II receptor antagonists
Propranolol 40 mg tab	Yes	Hypertension, management of angina pectoris, and control of most forms of cardiac arrhythmias	Beta blocker
Methyldopa 250 mg tab	Yes	Hypertension	Adrenergic receptor agonist
Amiodarone 200 mg tab	Yes	Arrhythmias	Antiarrhythmic drug
Bisoprolol 5 mg cap/tab	Yes	Hypertension and stable chronic angina	Beta blocker
Lovastatin 20 mg tab	No	Hypercholesterolemia/prevention of CVDs	HMG-CoA reductase inhibitors
Spironolactone 100 mg tab	Yes	Congestive heart failure	Potassium-sparing agents
Acetylsalicylic acid 75 mg tab	Yes	Secondary prevention of thrombotic cerebrovascular or cardiovascular disease	Antiplatelet drug

NEML, National Essential Medicines List.

### Data Collection

A data collection form was generated by using preprogrammed Excel workbook, available with the WHO/HAI manual (WHO, 2015). Trained data collectors visited the medicine outlets and recorded the medicines' availability by physically checking the stock while documenting the medicine prices. The data collection was started in Islamabad and subsequently collected in other regions under the supervision of four authors (AS, HS, ZS, and MS) assigned to specific regions. The data were obtained for OB and LPG of all the selected 18 CVD medicines. OB refers to the drug product that is sold by the original manufacturer which holds its intellectual property rights, and LPG refers to its lowest priced generic version available at the health facility being surveyed. In Pakistan, public hospitals provide free medicines to the patients, so from these facilities only medicines’ availability was recorded, while from private retail pharmacies data for both prices and availability of medicines were gathered.

### Data Entry and Analysis

The data were analyzed by using WHO/HAI preprogrammed workbook, Microsoft Excel, and IBM Statistical Package for the Social Sciences (SPSS) version 22.0. OANDA currency converter was used to convert the local prices to US dollars using the exchange rate on first day of data collection, that is, September 15, 2019 (where 1 USD = 156.56 PKR) (Currency Converter, Foreign Exchange Rates, OANDA, 2020).

#### Availability

Medicines’ availability was measured as the percentage of medicine outlets where the surveyed medicine was stocked on the day of data collection. WHO recommends that ideally the availability of 80% or more must be considered optimal for the Essential Medicines (EMs) (WHO, 2015). We described the availability of medicines according to the following criteria, used by many WHO/HAI surveys: <30%: very low, 30–49%: low, 50–80%: fairly high, and >80%: high (Rathish et al., 2017; Saeed et al., 2019). Associations between availability and other variables were analyzed using the binary logistic regression (available = 1, unavailable = 0). Beta coefficients with 95% confidence intervals (CI) were calculated after adjusting for medicine list (global = 0, supplementary = 1), sector (public = 0, private = 1), and brand (originator = 0, generic = 1). Multivariate logistic regression was used to estimate the association of key variables with the availability of medicines.

#### Prices

The medicine prices were measured as median price ratios (MPRs). The MPR is the ratio of median local unit price of a surveyed medicine to its international reference price (IRP). The supplier IRP of each medicine was obtained from the Management Sciences for Health (MSH) Drug Price Indicator Guide 2015. IRPs represent the procurement prices of multisource drug products offered by non-for-profit suppliers to the LMICs for the multisource products (International Medical Products Price Guide, 2020). According to WHO/HAI methodology, IRPs of a year before the survey should be used for MPR calculation. But the latest available IRPs were published in 2015, so we deflated our prices from 2019 to 2016 by 22%. The deflation factor (i.e., 22%) was calculated according to the consumer price indices (CPI) for drugs and medicines of Pakistan from 2016 to 2019 (Pakistan Bureau of Statistics, 2019). Similar to the other studies, this price adjustment method was adopted to make the comparison between local and international prices more reliable (Fang et al., 2013; Alefan et al., 2018; Song et al., 2018). In this study, in a private sector, a medicine's median MPR of more than 5 was considered as excessively priced. This criterion has previously been used by Babar et al. (2007). Although calculating the MPR is a credible method to compare medicine prices among countries, its interpretation still has some ambiguities due to varying medicine components, such as market size, market penetration, medicine pricing mechanisms, scale of economy, and taxation, among different countries. Multiple linear regression (beta coefficients, 95% CI) was used in the case of MPR (continuous outcome) to analyze associations between the variables (medicine list (global = 0, supplementary = 1) (originator = 0, generic = 1)) under investigation.

#### Affordability

Affordability was measured as the number of day's wages (NDWs) of a lowest paid government worker required to obtain the CVD medicines for a month. The standard treatment with a medicine was considered unaffordable if the patient has to spend more than one day's wage to purchase the treatment for a month, as described by the standard WHO/HAI methodology (WHO, 2015). The calculation of affordability was done using the following formula:

$$Number\;of\;days'wages\;required=\;\frac{Median\;unit\;price\;\times \;number\;of\;units\;needed\;per\;dose\;\times 30}{Wage\;of\;a\;lowest\;paid\;govt.\;\;worker\;per\;day}$$

where the salary of lowest paid government worker is 17,830 PKR (maximum) per month, that is, 594 PKR per day (with effect from July 2019). The affordability of all surveyed medicines was calculated as described in Table 5.

#### International Comparison of Affordability

To enhance the local relevance and international comparability, the medicines were selected according to the following criteria: their inclusion in the NEML, their potential impact on the burden of CVDs, and their frequency of inclusion in surveys by other LMICs. The affordability (NDWs) of OBs and LPGs of four CVD medicines (atenolol 50 mg, amlodipine 5 mg, captopril 25 mg, and simvastatin 20 mg) from the private sector of Pakistan was compared with affordability of these medicines in six other LMICs (see Table 6). The data on affordability of selected medicines in other LMICs (Afghanistan, Sudan, Lebanon, Egypt, India, and China) was retrieved from the global WHO/HAI database (World Bank Country and Lending Groups, World Bank Data Help Desk; Health Action International, 2020). Countries such as Afghanistan, Sudan, Lebanon, and Egypt fall under WHO's Eastern Mediterranean Region (EMR) (WHO, 2020b). As Pakistan is also under this region, these countries were used for comparison. Afghanistan also shares a long border with Pakistan, whereas India belongs to South East Asia Region (SEAR) of WHO but it is Pakistan's neighboring country and its medicine pricing and procurement system matches with that of Pakistan.

Pakistan also takes India as a reference country while setting the drug prices following external reference pricing mechanism (Drug Regulatory Autority of Pakistan, 2018a; Saeed, 2019). China is another neighbor located at northeastern border of Pakistan. One of the key reasons for selecting these countries for comparison, besides their economic status and geographical location, was the availability of data from the WHO/HAI methodology based surveys conducted in these countries. The latest available surveys were selected for data retrieval. The four selected medicines were most commonly surveyed among all the selected LMICs. Hence these four medicines were used for the comparison.

## Results

### Availability of Medicines

In public sector, the mean availability of both OB and LPG medicines was very poor, that is, only 25.5% and 30.4%, respectively. The situation was better in private sector, where the mean availability was 34.9% and 54.6% for LPGs and OBs, respectively (Supplementary Table S1). The regression analysis further demonstrated that the availability was significantly lower in the public sector as compared to the private sector (*p* < 0.001) (Table 3). Table 4 shows the mean percentage availability of each medicine, along with its categorization into very low, low, fairly high, and high availability, in both public and private sectors. None of the surveyed medicines had ideal availability of 80% in both public and private sectors. The OBs and LPGs of both lovastatin (5%) and hydrochlorothiazide-HCT (8%) were least available in the public sector. In the private sector, the OB HCT was absolutely unavailable and its LPG also had very poor availability (i.e., 25%). The overall availability of generics was significantly lower than the OBs (*p* < 0.001). The medicines from NEML showed better availability than the supplementary medicines (*p* = 0.019). Inter-regional variations with regards to medicine availability were also evident among the eight surveyed cities, where the public sector of Abbottabad and Peshawar had absolute non-availability of OBs on the day of data collection. The mean availability of OBs in the public sector ranged from 0% in Peshawar and Abbottabad to 53% in Karachi, while that of LPGs in the public sector ranged from 11% in Peshawar to 52% in Islamabad. The mean availability of OBs in the private sector ranged from 33% in AJK to 76% in Lahore, while that of LPGs in the private sector ranged from 10% in Quetta to 64% in both Karachi and Abbottabad (Supplementary Table S2).

**TABLE 3:** Regression model examining the effect of sector and medicine types and survey sectors on medicine availability and median price ratio.

Availability (binary logistic regression)							
	Coefficient	Standard error	Wald test	Significance	Exp (B)	95% confidence interval	
						Lower	Upper
Medicine list							
Global	1						
Supplementary	−0.260	0.111	5.471	0.019	0.771	0.620	0.959
Sector							
Public	1						
Private	1.094	0.087	159.79	0.000	2.985	2.519	3.535
Brand							
Originator brand (OB)	1						
Lowest price generic (LPG)	−0.463	0.085	29.635	0.000	0.630	0.533	0.744

Median price ratio (linear regression)							
	Coefficient	Standard error	Wald test	Significance		95% confidence	
						Lower	Upper
Medicine list							
Global	1						
Supplementary	−0.342	0.835	−0.410	0.685		−2.042	1.358
Brand							
Originator brand (OB)	1						
Lowest price generic (LPG)	−2.340	0.629	−3.717	0.001		−3.622	−1.058

**TABLE 4:** Individual medicines availability at healthcare facilities.

Medicine Name	Medicine type	%Availability in public sector%	Remarks	%Availability in private sector%	Remarks
Acetylsalicylic acid	Originator brand (OB)	5	Very low	25	Very low
Acetylsalicylic acid	Lowest price generic (LPG)	53	Fairly high	65	Fairly high
Amiodarone	OB	29	Very low	40	Low
Amiodarone	LPG	29	Very low	15	Very low
Amlodipine	OB	28	Very low	78	Fairly high
Amlodipine	LPG	38	Low	58	Fairly high
Atenolol	OB	40	Low	68	Fairly high
Atenolol	LPG	33	Low	63	Fairly high
Atorvastatin	OB	36	Low	65	Fairly high
Atorvastatin	LPG	50	Fairly high	63	Fairly high
Bisoprolol	OB	35	Low	68	Fairly high
Bisoprolol	LPG	23	Very low	40	Low
Captopril	OB	64	Fairly high	80	High
Captopril	LPG	29	Very low	30	Low
Digoxin	OB	50	Fairly high	60	Fairly high
Digoxin	LPG	29	Very low	10	Very low
Enalapril	OB	23	Very low	73	Fairly high
Enalapril	LPG	25	Very low	40	Low
Furosemide	OB	38	Low	73	Fairly high
Furosemide	LPG	25	Very low	18	Very low
Hydrochlorothiazide	OB	8	Very low	0	Very low
Hydrochlorothiazide	LPG	8	Very low	25	Very low
Losartan	OB	7	Very low	48	Low
Losartan	LPG	71	Fairly high	58	Fairly high
Lovastatin	OB	5	Very low	10	Very low
Lovastatin	LPG	5	Very low	28	Very low
Methyldopa	OB	28	Very low	65	Fairly high
Methyldopa	LPG	33	Low	10	Very low
Nifedipine retard	OB	21	Very low	38	Low
Nifedipine retard	LPG	29	Very low	20	Very low
Propranolol	OB	20	Very low	68	Fairly high
Propranolol	LPG	10	Very low	23	Very low
Simvastatin	OB	14	Very low	53	Fairly high
Simvastatin	LPG	29	Very low	43	Low
Spironolactone	OB	10	Very low	65	Fairly high
Spironolactone	LPG	33	Low	18	Very low

### Patient Prices in the Private Sector

**FIGURE 1:**
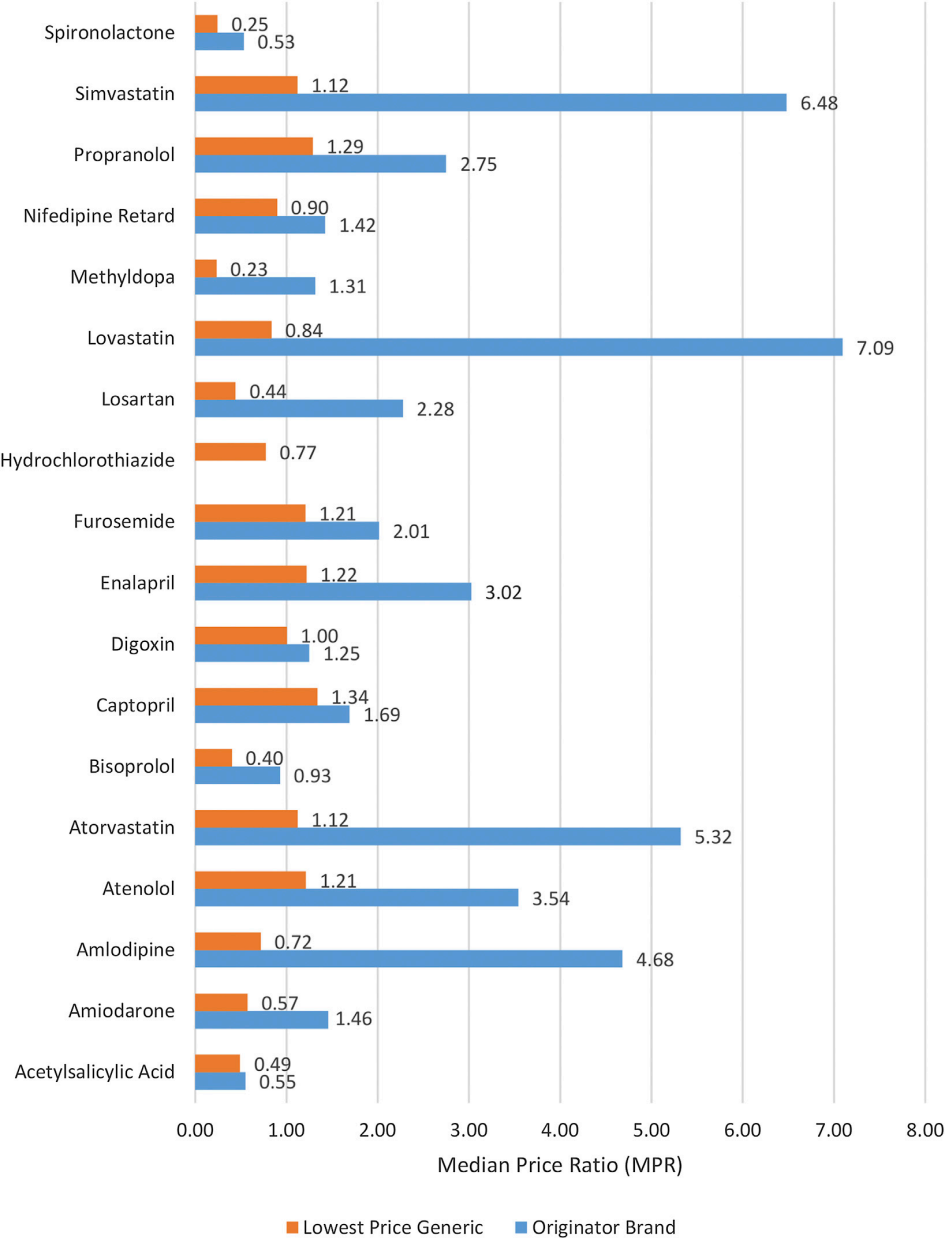
Median price ratios of both lowest price generics and originator brands in the private sector.

The medicine prices which patients had to pay (i.e., out of pocket payments) at private sector retail pharmacies were analyzed in terms of MPRs. For the public sector, the price analysis was not performed because medicines are provided without any charge to the patients. The MPR analysis included medicines with prices found for both types (OB and LPG) in pair (i.e., n = 17) (see Supplementary Table S3). The inflation-adjusted mean MPR was 2.72 and 1 for OBs and LPGs, respectively. In group-wise comparison, as shown in Table 3, the OBs had significantly higher prices than LPGs (*p* = 0.001). The differences between prices of global and supplementary list medicines were insignificant (*p* = 0.685). Three OBs had MPR above 5, the set benchmark, and were found excessively overpriced. For OBs, the top five highest MPRs were of lovastatin (7.09), simvastatin (6.48), atorvastatin (5.32), amlodipine (4.68), and atenolol (3.54). The inflation-adjusted MPRs of all LPG medicines were below 5, indicating tenuous affordability margin. The prices of seven LPG medicines were more than their respective IRPs. These medicines included captopril, with price 1.34 times the IRP, followed by propranolol (1.29), enalapril (1.22), atenolol (1.21), furosemide (1.21), atorvastatin (1.12), and simvastatin (1.12). The individual MPRs of all medicines (both OBs and LPGs) are shown in Figure 1 and Supplementary Table S3.

### Affordability

The specific cardiovascular conditions in which the surveyed medicines can be used are provided in Table 2. Table 5 showed the quantity of each medicine required for 30-day treatment course, median treatment price (MTP), number of days’ wages (NDWs) required, and the percentage of cost saved by using LPG instead of OB to obtain a standard treatment from a specific medicine. Data obtained demonstrated that CVD medicines were unaffordable, NDWs of more than 1, in the private sector with average NDWs of 6.4 and 2.2 for OB and LPG, respectively. Only spironolactone was found affordable with NDWs of 0.8 and 0.4 for both OB and LPG, respectively. Treatment courses with OBs of lovastatin (21.5), amlodipine (14.2), propranolol (12.5), simvastatin (9.8), and atorvastatin (8.1) were among the top five most unaffordable OBs. In case of LPGs, propranolol (5.8), captopril (4.1), furosemide (3.7), nifedipine retard (2.7), and lovastatin (2.5) were ranked higher in the list. We also estimated the cost that can be saved by using generics instead of brands by calculating the percentage difference of MTPs between OBs and LPGs. The results revealed that about 11%–88% of the treatment prices could be saved by switching to LPGs.

**TABLE 5:** Affordability of standard treatments in private retail pharmacies by lowest paid unskilled government worker.

Medicine with strength and dosage form	No. of units needed per treatment	Duration of treatment	Median treatment price (PKR)-OB	Median treatment price (PKR)-LPG	No. of days wages-OB	No. of days wages-LPG	% Difference between MTPs of OBs and LPGs (% of cost saved by using LPG)
Acetylsalicylic acid 75 mg tab	60	30	986.6	881.8	1.7	1.5	10.63
Amiodarone 200 mg tab	60	30	2624.8	1032.2	4.4	1.7	60.68
Amlodipine 5 mg tab	60	30	8427.1	1293.8	14.2	2.2	84.65
Atenolol 50 mg tab	30	30	3185.6	1091.7	5.4	1.8	65.73
Atorvastatin 20 mg cap/tab	30	30	4788.0	1010.2	8.1	1.7	78.90
Bisoprolol 5 mg cap/tab	60	30	1672.1	725.0	2.8	1.2	56.64
Captopril 25 mg cap/tab	60	30	3042.0	2411.3	5.1	4.1	20.73
Digoxin 0.25 mg tab	30	30	1124.9	903.6	1.9	1.5	19.67
Enalapril 5 mg tab	30	30	2720.3	1096.9	4.6	1.8	59.67
Furosemide 40 mg cap/tab	60	30	3620.5	2169.3	6.1	3.7	40.80
Hydrochlorothiazide 25 mg cap/tab	60	30	NA	1390.1	0.0	2.3	
Losartan 50 mg cap/tab	60	30	4096.8	792.9	6.9	1.3	80.64
Lovastatin 20 mg tab	60	30	12770.6	1503.5	21.5	2.5	88.22
Methyldopa 250 mg tab	90	30	3549.0	633.8	6.0	1.1	82.14
Nifedipine Retard 20 mg tab	60	30	2562.5	1618.4	4.3	2.7	36.84
Propranolol 40 mg tab	90	30	7419.8	3471.8	12.5	5.8	53.20
Simvastatin 20 mg cap/tab	30	30	5831.9	1008.2	9.8	1.7	82.71
Spironolactone 100 mg tab	30	30	478.4	220.8	0.8	0.4	53.80

### International Comparison of Affordability

The international comparison of the affordability of standard treatment with atenolol, amlodipine, captopril, and simvastatin showed that these drugs were highly unaffordable in Pakistan compared to Sudan, Lebanon, Egypt, India (Delhi), Afghanistan, and China (Shaanxi) (see Table 6). Overall, all four OBs and three out of four LPGs of selected CVD drugs were found highly unaffordable in Pakistan, which is alarming. The NDWs for OB atenolol were 5.4 in Pakistan and 5.2 in Sudan, while the rest of the countries had NDWs of less than 1. The OB amlodipine had NDWs of more than 1, unaffordable, in Pakistan (14.2), China (3.7), and Egypt (1.7) compared to other countries. The OB captopril was unaffordable in Pakistan (5.1) and Lebanon (1.3) and affordable in Egypt (0.8). OB simvastatin was unaffordable in all countries, ranging from 1.8 in Lebanon to 9.8 in Pakistan. The LPGs atenolol and amlodipine were found unaffordable only in Pakistan (1.8 and 2.2) among all the LMICs included for the comparison. The LPG captopril required the highest NDWs in Pakistan (i.e., 4.1) and was also unaffordable in Sudan (4.2) and India (1.1), while it was affordable in the rest of the LMICs. In case of LPG of simvastatin, it was highly unaffordable in Egypt (2.1), followed by Sudan (2), Pakistan (1.7), and Afghanistan (1.1), yet it was affordable in other LMICs included in the analysis.

**TABLE 6:** International comparison of affordability of standard treatments.

Country	Survey year	WB country income group	WHO region	Atenolol 50 mg tab		Amlodipine 5 mg tab		Captopril 25 mg cap/tab		Simvastatin 20 mg	
				OB	LPG	OB	LPG	OB	LPG	OB	LPG
Pakistan	2019	LMI	EMR	5.4	1.8	14.2	2.2	5.1	4.1	9.8	1.7
Sudan	2013	LMI	EMR	5.2	0.5	NA	0.8	NA	3.2	NA	2
Lebanon	2013	UMI	EMR	0.7	0.2	1	0.7	1.3	0.7	1.8	0.3
Egypt	2013	LMI	EMR	0.4	0.1	1.7	0.6	0.8	0.5	4.3	2.1
India	2011	LMI	SEAR	0.4	0.4	1	0.3	NA	1.1	2.2	0.6
Afghanistan	2011	LI	EMR	NA	0.2	NA	NA	NA	0.4	NA	1.1
China	2014	UMI	WPR	NA	NA	3.7	0.8	NA	NA	2.6	1.3

WB, World Bank; LMI, Lower Middle Income; UMI, Upper Middle Income; LI, Low Income; EMR, Eastern Mediterranean Region; SEAR, South East Asia Region; WPR, Western Pacific Region.

## Discussion

The results of this study underpinned the urgency to improve the availability of medicines for CVDs, particularly in the public sector. This is vital to ensure that the prescribed CVD medicines are available and affordable. It was surprising to see that none of the surveyed medicines met the ideal availability benchmark of 80% in both public and private sectors. Although these medicines are provided free of charge to the patients in the public sector, low availability (i.e., only 28%) compels the patients to purchase the medicines from the private sector. However, the availability at private retail pharmacies was also not up to the mark; that is, only 45% of the pharmacies had the surveyed medicines available on the day of data collection. The standard treatment affordability calculation in the private sector revealed that both OBs and LPGs of all selected medicines were not affordable for a lowest paid unskilled government employee. It should also be noted that about a quarter of Pakistan's population (i.e., 46 million) lives below the national poverty line and 4.5% of the people are unemployed, while those who are employed earn far less than the daily wage of a lowest paid government worker, which was used for treatment affordability calculation (Poverty and Equity Data Portal, 2020). In Pakistan, for everybody, access to public hospitals is almost free, with a paltry fee of rupees 20/-PKR (0.13USD), not limited to one consultation, where free medicines are provided after the consultation. However, for major surgeries and laboratory investigations, patients have to pay out of pocket. In private sector hospitals and pharmacies, the patients are supposed to pay for medical care including medicines. In 2016, in eight cities of Pakistan, a micro health insurance program was launched for the underprivileged citizens who intend to get treatment from private hospitals. So far, the program included 57 cities and enrolled approximately 7 million families, until February 2020. Although this is a good initiative by the Government of Pakistan, it does not specifically cover the medication cost incurred upon the patients. Besides, there is no particular financing scheme in place for the vast majority of the population who belong to middle income families and could hardly make their both ends meet. There is also lack of financial protection for the patients who obtain medicines from retail pharmacies.

According to our data, in the public sector, the LPGs had better availability than OBs; conversely, in the private sector, the availability of OBs was higher than that of LPGs. Since the overall availability was better in the private sector, it would be reasonable to interpret that patients had to purchase expensive OBs in most cases, compromising the affordability of patients. As the use of generic medicines can improve affordability for the masses, this also shows the need and potential to improve understanding of generic medicines in Pakistan's healthcare sector (Babar et al., 2007; Jamshed et al., 2009; Jamshed et al., 2011).

Similar trend regarding the availability of essential medicines was observed in previous surveys conducted in Lahore division of Pakistan, in 2016–17 and 2019 (Saeed et al., 2019; Saeed et al., 2020). There could be many reasons behind the poor availability of medicines, such as poor estimation of demands, delays in receiving the medication orders, and budgetary constraints. However, further in-house inspections are needed to determine the specific causes of poor availability. HCT was either unavailable or poorly available at both public and private health facilities, corroborating our previous study (Saeed et al., 2019) and a previous report on the availability of essential drugs, particularly HCTZ, for chronic diseases in six low and middle income countries including Bangladesh, Nepal, and Pakistan (Mendis et al., 2005). Furthermore, the poor availability of HCTZ can be attributed to the prescribing trends, as evident by a study from Karachi, Pakistan, suggesting that, among all the treatment options, thiazides were hardly prescribed to the patients, whereas beta blockers and angiotensin converting enzyme inhibitors were used more frequently. This could possibly explain the reason behind poor availability of HCT (Hussain et al., 2015).

Overall, the essential medicines listed in the National Essential Medicines List had better availability than the medicines listed in the supplementary list. Bazargani et al. did a secondary analysis of medicine's availability in the public and private sectors of 23 countries including surveys conducted utilizing WHO/HAI methodology. While doing this analysis, they categorized the medicines into essential and non-essential (not included into the NEML) and found that the availability of both types of medicines was suboptimal but the essential medicines had better availability than the non-essential medicines, corroborating our findings (Bazargani et al., 2014). Besides, the inter-regional comparison on medicine availability showed that OBs were more available in Karachi and Lahore. This could be due to better purchasing power and better economy of these big cities. Moreover, these cities are nerve centers of many multinational pharmaceutical companies; in addition, their offices and manufacturing units are based here too. Peshawar had relatively poor availability of selected medicines among all provincial capitals. The possible reason could be its relatively poor economy.

While doing the pricing analysis, only those medicines were included which had both OBs and LPGs available in the private sector. HCT was excluded as its OB was not available. This analysis was limited to the private sector only, because in the public sector, medicines are provided free of charge to the patients. The results revealed that the prices of selected CVD medicines were moderate to high in Pakistan, as the overall prices ranged from 0.23 to 7.09 times their respective IRPs. As expected, the regression analysis showed that the LPGs prices were lower than the OBs. A subsample of three OB medicines was highly expensive relative to the IRP. Seven LPG medicines were moderately expensive relative to the IRP, out of which, one LPG medicine (propranolol) exhibited substantial variation in unit price across the retail pharmacies, warranting further research to investigate reasons behind this variation. In Pakistan, in most cases the unit price of a medicine remained within a narrow range, probably because the maximum retail prices are fixed by the drug regulatory authority of Pakistan (DRAP) and also due to strong competition in domestic market. Moreover, the inspections by drug inspectors who are appointed by the government of Pakistan (responsible for monitoring of adherence to pricing policies by the retailers, distributors, and manufacturers) might have worked in this regard; nonetheless, they seldom monitor the stock control and inventory for both the OBs and the LPGs, which is purely driven by the prescribing trends.

Affordability of CVD medicines was also calculated in the private sector for both OB and LPG. The affordability calculation was made by considering number of days a lowest paid government worker would have to work in a month, for obtaining a standard course of treatment of a medicine. The results showed that treatment with both OBs and LPGs of CVD medicines were unaffordable. A study from Bangladesh also reported that CVD medicines were among most unaffordable essential medicines (Kasonde et al., 2019). OBs and LPGs of all drugs except one (spironolactone) were found to be unaffordable. In Pakistan, OOP expenditures are around 70% of the total healthcare expenditures. Not only are CVDs the most prevalent diseases among South Asian population, but also being a South Asian is considered a risk factor for CVD (Lozano et al., 2012; Khalid and Sattar, 2016). In this context, our data suggested that probably many CVD patients, especially living in poverty struck rural areas, had to forego the treatments, considering the extent of unaffordability of CVD medicines observed in this study. This could perhaps further lead to increase in CVD-related morbidity and mortality in Pakistan.

We also calculated the percentage difference between standard treatment prices of OBs and LPGs to estimate the cost savings after switching from OBs to generics. It was found that 11%–88% of the treatment price could be saved by switching from OBs to generics. This highlights the significance and need of generic prescribing, which could be one of the best possible solutions to reduce patient's OOP expenditures (Dixit et al., 2018).

The affordability of standard treatment with four CVD drugs in Pakistan was compared with that of six other LMICs. The LPGs were affordable in most of the countries, while OBs were unaffordable in most of the regions. The international comparison on the affordability of medicines suggested that the treatment for CVDs is markedly unaffordable in Pakistan, both OBs and LPGs of selected CVD medicines, compared to the other six LMICs. However, the small sample size of CVD medicines for this comparison limits the generalizability of the results.

There could be several implications for policy makers that are needed to be considered while devising medicine pricing policies. NEML-based procurement system must be ensured at least in public sector hospitals. Rules must be devised to maintain a minimum stock level of essential medicines in the private sector as well. Keeping in view the high prevalence of chronic diseases such as CVDs and diabetes, the NEML could be bifurcated into main groups of medicines (i.e., medicines for chronic and acute diseases). It should be also mandated for every public sector hospital to procure certain number of medicines from both groups. This could help improving the availability of medicines for chronic diseases. Moreover, generic prescribing must be mandated to reduce the burden on patients' pocket. But there could be several limitations in the implementation of generic prescribing in Pakistan's healthcare settings. There are no regulations that prohibit a pharmacist from substituting branded drugs with generic drugs but like many other LMICs, high perverse incentives from the multinational companies along with pressure from medical representatives lead to malpractice of brand drug prescribing and dispensing (Jamshed et al., 2012). A study conducted in Peshawar, Pakistan, reported a very high number of brands in the prescriptions, as compared to generics, indicating brand prescribing monopoly patronized by the doctors, co-influenced by pharmaceutical companies due to overt promotion of their brands knowledge (Raza et al., 2014). Another study conducted in Lahore, Pakistan, reported that the majority of the medical and pharmacy students had doubts on the bioequivalence of generic drugs to their respective OBs. These issues highlight the pressing need for training of medical and pharmacy students, practitioners, and pharmacists regarding dispensing and prescribing of generic drugs. Besides, interventions should be made to educate the public about availability, efficacy, and cost effectiveness of generic drugs (Asif et al., 2018). Innovative financing mechanisms that support the sale of essential medicines for chronic diseases at private retail pharmacies must be introduced.

Mechanism for regular monitoring and reporting of availability and prices of essential medicines must be devised. The drug inspectors or a third party regulator could be given the responsibility of monitoring the medicine price and availability. A mobile application, “WHO Essential Medicines and Health Products Price and Availability Monitoring (WHO EMP MedMon)” developed by WHO, could also be used for pricing data collection and analysis. This application is based on standard WHO/HAI methodology (WHO, 2019). The local production of essential medicines for chronic diseases must be promoted by subsidizing raw material. Implementation of differential pricing mechanism and tax exemptions for essential medicines could be one of the rational options. The medicines and public sector hospitals could be partially subsidized while giving priority to the medicines used for chronic/most prevalent diseases. Pakistan should also learn from the pricing strategies being employed by other LMICs.

Our study also had some limitations as follows: only those CVD medicines were included in the survey which had Management Sciences for Health International Reference Prices (MSH IRP) available. This is for the sake of MPR calculation, as required by standard WHO/HAI methodology. This means that medicines with specific strength and dosage form were included. This could have resulted in underestimation of the availability of some medicines because the surveyed facility could have stocked the other dosage form or strengths of the surveyed medicines on the day of data collection. The MPRs were calculated in this study by using 2015 MSH IRPs because the latest MSH IRPs were not available. However, we have deflated the prices using the CPI in order to make the MPR calculation reliable. To resolve this issue, the HAI experts suggest avoiding calculating it and rely on median unit prices (MUPs) only. So, we have also calculated and presented the MUPs. The affordability calculations were made for each medicine separately, whereas most of the patients with chronic diseases have co-morbid conditions that require multiple medications at a time. This would further increase the number of CVD patients who could not afford these medicines. It is a cross-sectional study and does not reflect the long-term availability and affordability of medicines. So, there is a need to perform longitudinal studies in this regard. Since the study regions and medicine outlets were selected on the basis of WHO/HAI methodology, most of the major cities in Pakistan got selected from each province. Nonetheless, only a few rural and peri-urban medicine outlets were surveyed, limiting the generalizability of results to rural regions. Lastly, the international comparison of medicines affordability in Pakistan with that of other LMICs included the surveys which were a few years apart. This might have affected the reliability of the comparison. However, it does provide a useful evidence to the policy makers about the difference of affordability of CVD merdicines in these countries.

## Conclusion

Our study has shown poor availability of CVD medicines in public sector hospitals, forcing the patients to pay out of pocket for the purchase of medicines from private sector retail pharmacies. Although the availability of medicines was better in the private sector, it was below the standard benchmark defined by WHO. When compared to IRPs, the OBs were found to be high priced in case of a number of medicines. However, the prices of LPGs were somewhat reasonable in private sector retail pharmacies necessitating to further improve the use of generic medicines. The standard courses of treatment with both the OBs and LPGs were not affordable for a lowest paid unskilled government worker. Overall, the majority of the selected CVD medicines were found quite unaffordable in Pakistan when compared with other LMICs. Among possible solutions, the implementation of generic prescribing is imperative. Moreover, policies should be devised to enforce NEML-based procurement system, promote the local production of CVD medicines and regular and systematic monitoring of medicines' prices and availability, implement generic prescribing and dispensing, and encourage regular research in this area.

## Data Availability

The raw data supporting the conclusions of this article will be made available by the authors, without undue reservation.
