# Supplementary material for: Access to Essential Cardiovascular Medicines in Pakistan: A National Survey on the Availability, Price, and Affordability, Using WHO/HAI Methodology
Source: Front Pharmacol. 2021 Jan 25;11:595008. doi: 10.3389/fphar.2020.595008 (PMC7941209; doi:10.3389/fphar.2020.595008)
Supplement: Supplementary file 1 [file table1.docx]

**Supplementary Material**

**Table A.** Mean % availability and median MPRs of different categories of medicines, at both public and private healthcare facilities.

|  | **Public Sector (n=40 outlets)** | | | | | |
| --- | --- | --- | --- | --- | --- | --- |
|  | **All medicines**  **(n=18)** | | **NEML medicines only (n=15)** | | **Global list medicines (n=3)** | |
|  | **OB** | **LPG** | **OB** | **LPG** | **OB** | **LPG** |
| **Mean % Availability (±SD)** | 25.5  (16.0) | 30.4  (16) | 23.8  (14.0) | 31.0  (16.0%) | 37.9  (25.0) | 26.5  (3.5) |
|  | **Private Sector (n=40 outlets)** | | | | | |
|  | 54.6  (23.5) | 34.9  (19.6) | 55.7  (21.5) | 34.5  (20.2) | 66.7  (13.8) | 37.5  (6.6) |
|  | **All medicines**  **(n=17*)** | | **NEML medicines only (n=14*)** | | **Global list medicines (n=3)** | |
|  | **OB** | **LPG** | **OB** | **LPG** | **OB** | **LPG** |
| **Mean MPR** | 2.7 | 1.0 | 2.3 | 0.7 | 3.0 | 1.0 |
| **Maximum MPR** | 7.0 | 1.3 | 6.4 | 1.2 | 6.4 | 1.3 |
| **Minimum MPR** | 0.5 | 0.2 | 0.5 | 0.2 | 0.9 | 0.4 |

* Analysis includes only medicines with prices found for both types in pair.

**Table B. Mean availability of originator brands (OBs) and lowest price generics (LPGs) in public and private sectors of eight cities of Pakistan.**

| **City** | **Mean % availability in public sector** | | **Mean % availability in private sector** | |
| --- | --- | --- | --- | --- |
|  | **OB** | **LPG** | **OB** | **LPG** |
| **Abbotabad** | 0 | 20 | 74 | 64 |
| **Karachi** | 53 | 34 | 74 | 64 |
| **Lahore** | 48 | 20 | 76 | 46 |
| **Islamabad** | 16 | 52 | 52 | 41 |
| **Bahawalpur** | 16 | 18 | 38 | 37 |
| **Peshawar** | 0 | 11 | 48 | 28 |
| **Azad Kashmir** | 24 | 11 | 33 | 11 |
| **Quetta** | 15 | 47 | 67 | 10 |

**Table C. Adjusted median price ratios (MPRs) of both originator brands (OBs) and lowest price generics (LPGs) in private sector retail pharmacies.**

| **Medicine Name** | **Median Unit Prices of OBs (PKR)** | **Median Unit Prices of LPGs (PKR)** | **Adjusted MPR of OBs** | **Adjusted MPR of LPGs** |
| --- | --- | --- | --- | --- |
| Acetylsalicylic Acid | 1.6 | 1.43 | 0.55 | 0.49 |
| Amiodarone | 22.06 | 8.675 | 1.46 | 0.57 |
| Amlodipine | 14.59 | 2.24 | 4.68 | 0.72 |
| Atenolol | 7.47 | 2.56 | 3.54 | 1.21 |
| Atorvastatin | 112.8 | 23.8 | 5.32 | 1.12 |
| Bisoprolol | 16.71 | 7.245 | 0.93 | 0.40 |
| Captopril | 8.2 | 6.5 | 1.69 | 1.34 |
| Digoxin | 2.49 | 2 | 1.25 | 1.00 |
| Enalapril | 6.2 | 2.5 | 3.02 | 1.22 |
| Furosemide | 2.42 | 1.45 | 2.01 | 1.21 |
| Losartan | 51.67 | 10 | 2.28 | 0.44 |
| Lovastatin | 21.83 | 2.57 | 7.09 | 0.84 |
| Methyldopa | 8.4 | 1.5 | 1.31 | 0.23 |
| Nifedipine Retard | 5.7 | 3.6 | 1.42 | 0.90 |
| Propranolol | 3.74 | 1.75 | 2.75 | 1.29 |
| Simvastatin | 67.1 | 11.6 | 6.48 | 1.12 |
| Spironolactone | 10.4 | 4.8 | 0.53 | 0.25 |

**Figure A. A macrograph depicting sampling of medicine outlets within a survey area/city.**

**One survey Area/City**

Within 10 Km

Within 10 Km

Within 10 Km

Within 10 Km

Within 10 Km

Retail Pharmacy

Retail Pharmacy

Retail Pharmacy

SAs within 3 hours drive from survey anchor

Retail Pharmacy

Retail Pharmacy

Where, SU denotes the Survey Unit.
